# Oral health-related quality of life and survival analysis after preventive and restorative treatment of molar-incisor hypomineralisation

**DOI:** 10.1038/s41598-024-51223-3

**Published:** 2024-01-08

**Authors:** Caroline Sekundo, Marina Jung, Clara Muscholl, Cornelia Frese

**Affiliations:** Department of Conservative Dentistry, Clinic for Oral, Dental and Maxillofacial Diseases, University Hospital Heidelberg, Heidelberg University, Im Neuenheimer Feld 400, 69120 Heidelberg, Germany

**Keywords:** Paediatric research, Outcomes research

## Abstract

The aim of this study was to assess the impact of molar-incisor hypomineralisation (MIH) on oral health-related quality of life (OHRQoL) in children and adolescents, including information on restorative care, tooth sensitivity, as well as sociodemographic factors. Thirty-five patients aged between 7 and 17 years underwent a comprehensive oral examination. Severity of MIH was graded using the MIH Treatment Need Index (MIH-TNI), OHRQoL using the Child Oral Health Impact Profile (COHIP-19). Clinical quality of restorations was assessed according to modified FDI-criteria, tooth sensitivity using the Schiff Cold Air Sensitivity Scale (SCASS). The mean age was 11.3 ± 3.0 years, 34% were female. On average, 6.9 ± 2.8 teeth were affected, 62,9% had hypersensitive teeth (SCASS ≥ 1). Eighty-nine percent of patients had received restorative care, with a mean of 3.3 ± 2.1 teeth restored, most often with composite, followed by fissure sealing. Nine percent of restorations failed by the FDI-criteria. Mean estimated survival times for success were 4.9 years (95% CI 3.5; 6.2) and 5.6 years (95% CI 5.0; 6.3) for fissure sealants and composite restorations, respectively. The mean COHIP-19 score was 64.3 ± 8.2 (max. possible score = 76). A higher severity of MIH-TNI correlated significantly with impaired OHRQoL (r_s_ = − 0.38, p = 0.013). However, this was not mirrored in multiple regression analysis. Despite the high rate of restorative treatment with an acceptable failure rate, OHRQoL is reduced in children with MIH. Many teeth affected by MIH remain sensitive. Further studies are needed to assess the benefits of different restorative options.

## Introduction

Molar-incisor hypomineralisation (MIH) is characterized by enamel defects of one or more first permanent molars with our without involvement of the permanent incisors^1^. To date, the aetiology of the disease remains unclear. According to the current state of research, a combination of systemic and external influences is probable^2,3^. Many factors have been suggested and a variety of associations has been reported, such as maternal illness, medication use during pregnancy, complications at birth or early childhood illness^3^. However, the quality of evidence regarding these associations remains low^3^. Reports on the prevalence of MIH vary widely, ranging from 2.4 to 40.2%^4^. A more recent meta-analysis from 2017 found an average prevalence of 14.2% worldwide^5^. These variations in reported prevalence notwithstanding, MIH is one of the most common non-carious diseases of the dental hard tissues in childhood^6^.

The burden of disease is high, both in terms of dental clinical findings and in terms of the children's quality of life. The mechanical strength of teeth affected by MIH can be significantly reduced. In addition, the affected teeth have an increased susceptibility to caries^7^. They also often present a major challenge during dental treatment in terms of adequate restorative care^8^. So far, the therapeutic options available do not offer a curative approach and are exclusively symptom-oriented. These include caries-preventive interventions such as concentrated fluoride applications and fissure sealants, the applications of preparations based on casein phosphopeptide and amorphous calcium phosphate^9^, as well as direct restorations and prefabricated steel crowns. For teeth that cannot be preserved, extraction followed by orthodontic treatment can be considered^10,11^.

The oral health-related quality of life of children and adolescents can be impacted by the fact that the affected teeth are often extremely sensitive^12^. Children affected by MIH often suffer from severe discomfort during food intake and during tooth brushing, which can result in inadequate oral hygiene and limitations to daily life^13^. Moreover, anaesthesia may be difficult at times^14^, and the young age of many patients and difficult restoration of the affected teeth result in a large number of necessary dental interventions^15^. These factors can also lead to an increased incidence of dental fear and anxiety^15^. Research regarding the impact of MIH on oral health-related quality of life has only recently come into the focus of scientific interest^16–18^, and few have yet assessed restorative treatment as a potential influencing factor^19^. The studies to date show that MIH, and in particular the location of the affected teeth in the oral cavity, have a significant impact on oral health-related quality of life in children and adolescents^20,21^. It is for this reason that we aimed to assess the impact of MIH on OHRQoL in children and adolescents, including information on restorative care, tooth sensitivity, as well as demographic factors such as age and sex. The following null hypotheses were formulated: (1) OHRQoL is not impaired in children whose teeth affected by MIH have been treated. (2) In case of restorative treatment, the survival rate of restorations is not reduced compared to survival rates of restorations in MIH-free teeth.

## Materials and methods

The study was structured in two parts to address the study hypotheses: (1) a cross-sectional study of OHRQoL in children and adolescents treated for MIH (the primary outcome being the association between OHRQoL and MIH severity, secondary outcomes being associations with caries experience, tooth sensitivity, oral hygiene parameters, restoration type, age and sex) and (2) a retrospective analysis of survival rates of restorations placed in this patient group. Patients who had been treated for MIH at the Department of Conservative Dentistry, Heidelberg University Hospital, between 2015 and 2022, were included. The study was approved by the local ethical commission (S-550/2021) and registered with the German Clinical Trials Register (DRKS 00030206). The following inclusion criteria had to be met: the patient (1) is between the age of 7 and 17, (2) can maintain good oral hygiene at home and (3) is diagnosed with MIH.

Initial search of patient records resulted in a total of 45 underage patients that had presented with MIH in our department. All 45 patients were eligible and were invited to participate in a cross-sectional examination. Reasons for non-response were no contact established/ no interest (n = 2), the patient having moved too far away (n = 3) and illness (n = 5). The non-response rate was thus 22%. Thirty-five patients agreed to participate and underwent a comprehensive oral examination. Written informed consent was obtained from all patients and parents/legal guardians.

During the clinical examination, a detailed medical history was taken, an intraoral examination was carried out and photographs were taken. The severity of MIH was graded using the MIH Treatment Need Index (MIH-TNI)^22,23^ (for details, see Table A.1 in the Appendix). The clinical quality of restorations placed in teeth affected by MIH was assessed according to modified FDI-criteria^24,25^. These include aesthetic, functional and biological parameters and are expressed with five scores, three for acceptable (score 1–3) and two for non-acceptable (4–5). Tooth sensitivity was assed using the Schiff Cold Air Sensitivity Scale (SCASS)^26^. Caries experience was recorded by means of the DMF-T according to WHO basic methods^27^. Moreover, the Plaque Control Record (PCR)^28^ and Gingiva Bleeding Index (GBI)^29^ were measured. The clinical parameters were recorded by two calibrated examiners under a conventional operating light source (SIROLUX F, Sirona Dental Systems GmbH, Bensheim, Germany) and with the help of magnifying glasses (2.5 old). The clinical examinations were conducted by two investigators (M.J., C.M.) who had not carried out any of the treatments that were evaluated. Calibration regarding the FDI criteria and MIH-TNI was performed using standardized photographs.

The assessment of OHRQoL was performed by means of the 19-item German version of the Child Oral Health Impact Profile (COHIP-19)^30^. The COHIP has shown strong reliability, validity and sensitivity to change, making it a valuable tool in both clinical dentistry and research for evaluating the impact of oral health on the well-being and daily life of young patients^31^. The child was asked to complete the questionnaire independently (without parental help); if there were any difficulties in understanding the questionnaire, the study doctor provided explanations. For each of the 19 questions, the patients were asked how frequently they had experienced the positive or negative impact during the past 3 months. Responses were graded on a five-point Likert-type scale (0 = never, 1 = almost never, 2 = sometimes, 3 = fairly often, and 4 = almost all of the time). Scorings of negatively worded items were reversed^30,31^. The questionnaire had three subscales: oral health—well-being (5 items), functional well-being (4 items) and social/emotional, school and self-image subscale (10 items). The overall summary score ranged from 0 (worst OHRQoL) to 76 (best OHRQoL) (for further details, see Table A.2 in the Appendix).

Due to the small number of restorative materials other than fissure sealants and composite used on MIH teeth, Kaplan–Meyer survival analysis was performed only for those restorations. Participants’ records were studied for information on the time of the restorations’ fabrication and any previous repair or renewal. Any failure by the FDI criteria (rating in categories 4 or 5) during the clinical examination or irreparable loss that required the fabrication of a new restoration was recorded as “failure” (F). Less serious unfavourable events that were reparable up to three times were defined as “survival with repair” (SR). Restorations that had to be repaired more than three times during the observation period were also registered as failure. Restorations without any unfavourable event were classified as “success” (S).

Statistical analyses were carried out with SPSS, version 24.0^32^. This is an explorative study and a p value of 0.05 or less was considered as statistically significant. Characteristics of the study population are presented by means of descriptive statistics. Mean ± SD of continuous variables and proportion and frequency of categories of factor variables are reported. To assess the normality of the distribution of COHIP-19 scores, both the Kolmogorov–Smirnov test with Lilliefors significance correction and the Shapiro–Wilk test were conducted. The Kolmogorov–Smirnov test yielded a statistic of 0.174 with a p value of 0.009, while the Shapiro–Wilk test showed a statistic of 0.932 and a p value of 0.033. These results indicate a significant deviation from a normal distribution. Consequently, non-parametric methods were employed for further analyses. Specifically, Spearman rank correlation was utilized to assess the relationships between OHRQoL and various clinical parameters. Correlation coefficients were appraised according to Chan 2003^33^. A multiple linear regression analysis was performed to explore the combined effect of the identified factors on OHRQoL The success analysis was done according to the Kaplan–Meier method^34^.

### Ethical approval

The study was conducted in accordance with the Declaration of Helsinki, and approved by the Ethics Committee of the medical faculty of Heidelberg University (protocol code S-550/2021, date of approval 22.07.2021).

### Informed consent

Informed consent was obtained from all subjects involved in the study.

## Results

Characteristics of the study population are summarized in Table 1. The mean age of participants was 11.3 ± 3.0 years. The majority of participants did not have any general diseases, allergies or used any medication.

**Table 1:** Characteristics of the study population.

Variable	Children and adolescents with MIH (n = 35)		n (%)
Age (years)	Mean (SD)	11.3 (3.0)	
	Min–Max	7–17	
Sex	Male		23 (65.7)
	Female		12 (34.3)
General diseases	None		25 (71.4)
	Coagulation disorders		3 (8.6)
	Asthma		4 (11.4)
	ADHD		1 (2.9)
	VACTERL association		1 (2.9)
	Epilepsy		1 (2.9)
Medication	None		32 (91.4)
	Contraceptives		1 (2.9)
	Iron supplements		1 (2.9)
	Macrogol		1 (2.9)
Allergies	Yes		6 (17.1)
	No		29 (82.9)
DMF-T	Mean (SD)	2.9 (2.1)	
	Min–Max	0–11	
Number of permanent teeth affected by MIH	Mean (SD)	6.9 (2.8)	
	Min–Max	2–12	
Number of primary teeth affected by MIH	Mean (SD)	0.6 (1.1)	
	Min–Max	0–4	
Most severe MIH-TNI Code	Code 1		3 (8.6)
	Code 2a		2 (5.7)
	Code 2b		4 (11.4)
	Code 2c		5 (14.3)
	Code 3		0
	Code 4a		3 (8.6)
	Code 4b		10 (28.6)
	Code 4c		8 (22.9)
Restorative treatment of MIH teeth	Yes		31 (88.6)
	No		4 (11.4)
Most severe SCASS Code	Code 0 (no response to air stimulus)		13 (37.1)
	Code 1 (slight response, no request for discontinuation)		15 (42.9)
	Code 2 (strong response, request for discontinuation or movement)		4 (11.4)
	Code 3 (stimulus considered painful, request for discontinuation)		3 (8.6)

**Figure 1:**
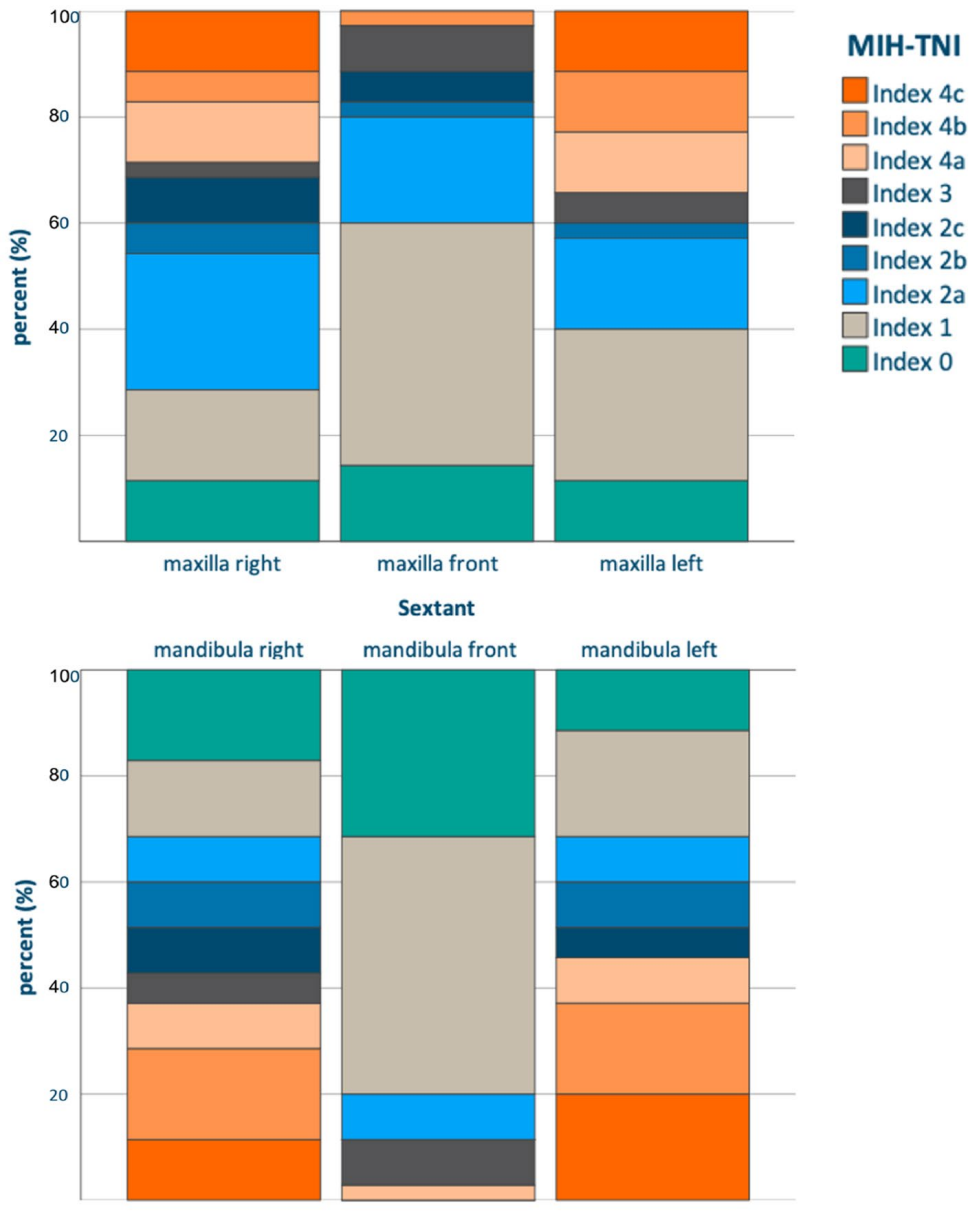
Distribution of MIH-severity according to the MIH-TNI Index.

On average, 6.9 ± 2.8 permanent teeth were affected, in contrast, only few primary teeth with MIH could be registered. Regarding the distribution of MIH, molars were more severely affected than anterior teeth, particularly in the mandible (Fig. 1). For more than half of participants, the most severe MIH-TNI Code was 4b or 4c, i.e. defects with hypersensitivity, exceeding 1/3rd of the enamel surface.

Sixty-three percent of participants had a SCASS score ≥ 1 and thus hypersensitive teeth. Eighty-nine percent of patients had received some form of restorative care on MIH teeth, with a mean of 3.3 (SD = 2.1) teeth restored.

**Figure 2:**
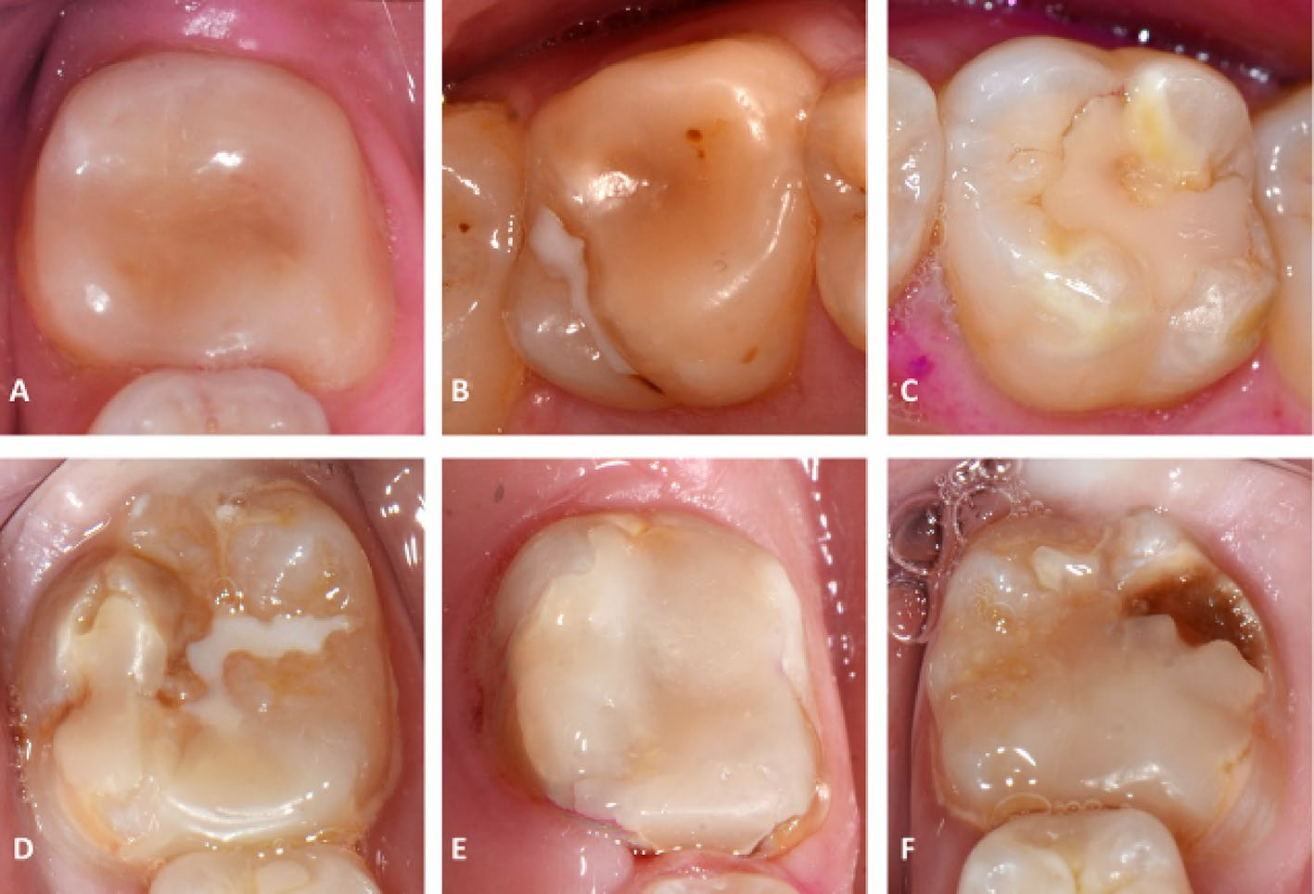
Examples of composite restorations on MIH-affected teeth. (**A**–**C**) Clinically acceptable composite restorations (clinically very good—clinically acceptable depending on the domains of the modified FDI criteria); (**A**) lower left first molar, follow-up 1.5 years; (**B**) upper right first molar, follow-up 7 years; (**C**) upper LEFT first molar, follow-up 7 years; (**D**–**F**) clinically inacceptable composite restorations (clinically unsatisfactory); (**D**) lower right first molar, follow-up 4 years, unsatisfactory marginal adaptation; (**E**) lower left first molar, follow-up 4.5 years, unsatisfactory marginal adaptation and presence of caries at restoration margin; (**F**) lower left first molar, follow-up 4 years, unsatisfactory marginal adaptation and presence of caries at restoration margin.

No restorative treatment was required in 45.6% of cases (n = 110); the teeth were monitored regularly. Most teeth were treated by means of composite restorations (31.1%, n = 75) or fissure sealants (12.9%, n = 31). Of the direct composite restorations (n = 75), 29.3% (n = 22) were applied over the entire tooth surface, similar to a composite crown. For further details on the distribution of other treatment measures performed, please see Fig. A.1 in the Appendix. Nine percent of restorations failed by the modified FDI-criteria at the time of the cross-sectional clinical examination. The most frequent reasons for the failure of composite restorations were insufficient marginal adaptation (n = 4), insufficient tooth integrity (n = 5) and recurrence of caries (n = 3). Examples of successful and failed restorations are illustrated in Fig. 2. All fissure sealings were acceptable at the time of examination.

The mean summary COHIP-19 score was 64.3 ± 8.2. All of the three subscales were impaired (oral health—well-being, functional well-being, social/emotional, school and self-image subscale), however, “oral health—wellbeing” was most impaired (Table 2). A higher severity of MIH-TNI demonstrated a fair correlation with impaired OHRQoL (r_s_ = − 0.38, p = 0.013), as did a higher DMF-T (r_s_ = − 0.36, p = 0.017) and SCASS (r_s_ = − 0.29, p = 0.043). No correlation with OHRQoL could be found for sex (p = 0.32), age (p = 0.55), the presence of a general disease (p = 0.15), medication (p = 0.58), allergies (p = 0.45), GBI (p = 0.07), PCR (p = 0.10) or the number of teeth having received any specific type of restoration (0.13 < p < 0.45). Table 3 shows the OHRQoL in relation to the most severe MIH-TNI diagnosis.

**Table 2:** COHIP-19 item, subscale and summary scores (n = 35).

Content	Mean (SD)	Range
**Oral health—well-being**		
1. Had pain in your teeth/toothache	3.1 (0.9)	1–4
2. Had discolored teeth or spots on your teeth	2.2 (1.6)	0–4
3. Had crooked teeth or spaces between your teeth	2.5 (1.5)	0–4
4. Had bad breath	3.1 (1.0)	1–4
5. Had bleeding gums	2.8 (1.3)	0–4
Oral health subscale (max. score 20)	13.7 (4.3)	3–20
**Functional well-being**		
6. Had difficulty eating foods you would like to eat	3.3 (1.1)	0–4
7. Had trouble sleeping	3.9 (0.5)	2–4
8. Had difficultly saying certain words	3.9 (0.2)	3–4
9. Had difficulty keeping your teeth clean	3.3 (1.2)	0–4
Functional well-being subscale (max. score 16)	14.4 (2.4)	8–16
**Social/emotional, school and self-image**		
10. Been unhappy or sad	3.5 (1.0)	0–4
11. Felt worried or anxious	3.7 (0.6)	2–4
12. Avoided smiling or laughing with other children	3.8 (0.6)	2–4
13. Felt that you look different	3.8 (0.5)	2–4
14. Been worried about what other people think about your teeth, mouth, or face	3.6 (1.0)	0–4
15. Been teased, bullied, or called names by other children	3.8 (0.6)	1–4
16. Missed school for any reason	3.7 (0.7)	1–4
17. Not wanted to speak/read out loud in class	3.9 (0.2)	3–4
18. Been confident	3.1 (1.4)	0–4
19. Felt that you were attractive (good looking)	2.9 (1.3)	0–4
Social/emotional, school and self-image subscale (max. score 40)	35.8 (4.3)	22–40
**Summary score (max. score 76)**	64.3 (8.2)	45–76

Scores of negatively worded items (all items except #18 and #19) were reversed before analyses.

**Table 3:** COHIP-19 mean summary score for MIH-affected patients (n = 35).

	Mean (SD)
All	64.3 (8.2)
Female	66.0 (8.1)
Male	63.4 (8.3)
Max. MIH-TNI 1 (n = 3)	71.0 (4.6)
Max. MIH-TNI 2 (n = 11)	67.9 (4.5)
Max. MIH-TNI 4 (n = 21)	62.3 (10.3)

Based on the significant correlations observed in the bivariate analysis, a multiple linear regression was conducted, including the following independent variables: Max. MIH-TNI Code, DMFT, dmft and max. SCASS Code. The model accounted for 39.8% of the variance (R^2^ = 0.398) in the COHIP-19 summary score and was statistically significant, F(4, 20) = 3.308, p = 0.031. However, when examining the individual predictors within the model, none of the variables showed a significant impact at the 5% level (see Table A.3 in the Appendix for further details).

**Figure 3:**
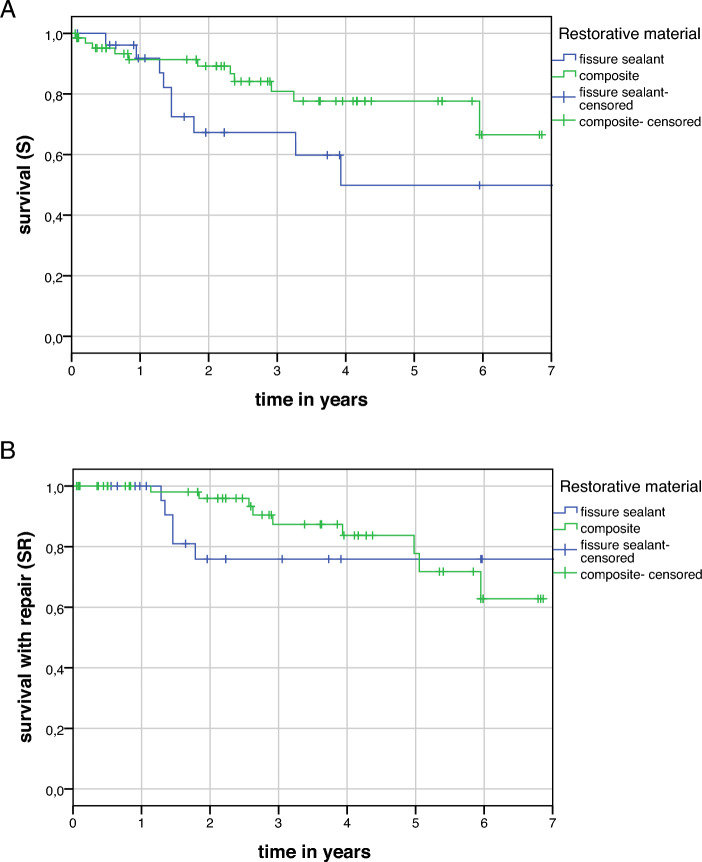
Kaplan–Meier survival curve of 27 fissure sealings and 67 composite restorations on teeth affected by MIH (**A**) complete success (**B**) including success with repair.

Regarding the survival analysis, the mean follow-up time was 2.9 (SD = 2.3) years for all types of restorations. According to the definition, complete success (S) was achieved in 82/102 (80.4%) of all restorations at the last follow-up. Success with repair (SR) was achieved in 88/102 (86.3%) of cases. Kaplan–Meyer survival analysis was performed only for fissure sealants and composite due to the small number of other restorations. Information on 27 fissure sealings and 67 composite restorations were sufficiently documented in patients’ records and could be included in the survival analysis. The mean estimated survival times for complete success (S) were 4.9 years (95% CI 3.5; 6.2) and 5.6 years (95% CI 5.0; 6.3) for fissure sealants and composite restorations, respectively. For success with repair (SR), the corresponding mean estimated survival times were 7.9 years (95% CI 6.3; 9.4) and 5.9 years (95% CI 5.3; 6.4) (Fig. 3a + b).

## Discussion

In this cross-sectional examination and retrospective analysis of children and adolescents treated for MIH mainly by means of preventive and restorative interventions, clinical quality and survival rates of these restorations were determined. Moreover, the severity of the disease significantly influenced OHRQoL, even after restorative treatment of affected teeth. A similar correlation could be found for the DMF-T and SCASS. This is coherent, seeing as many children had received restorations or extractions due to their MIH, thus increasing their DMF-T score, and that most had MIH forms with increased tooth sensitivity, thus also mirrored in the SCASS. Moreover, patients with MIH also have an increased susceptibility to caries^7,35^.

These findings are in line with previous studies, who have also shown that impairment of OHRQoL can be caused by MIH^20,21^. In 2016, Dantes-Neta et al. reported a negative influence of MIH on oral well-being and functional limitations using the Child Perception Questionnaire in a cross-sectional study among 594 Brazilian schoolchildren^18^. The latter questionnaire was also used in later studies among Brazilian and Mexican schoolchildren with similar set-up^16,17,36^. As the CPQ in its age-dependent variants is broadly similar to the COHIP^37^, these results seem transferable. In contrast to the present study, however, MIH severity was graded depending on the appearance and size of defects^16–18,36^, without taking into account hypersensitivity, as is done by means of the MIH-TNI classification. However, this is an important factor, as the results of the present study show differences in OHRQoL between categories 2 and 4 of the MIH-TNI, which do not differ in clinical appearance, but only in clinical symptoms. To date, only one other study has compared the MIH-TNI to OHRQoL, reporting a similar relationship^21^.

Regarding the OHrQoL domains of the COHIP instrument, the 'oral health well-being' domain, assessing the impact of oral health on physical comfort, appearance, and overall health, showed the most impairment. This was largely attributed to discoloured teeth, a visible symptom of MIH. The 'functional well-being' domain, which encompasses the influence of oral health on basic functions like eating, speaking, and sleeping, was primarily affected due to difficulties in eating and cleaning teeth, likely due to hypersensitivity^12^. These findings align with previous research^13^. In the social/emotional domain, children with MIH often exhibited reduced confidence and did not always feel attractive. The psychosocial impact of dental issues is noteworthy, as bullying related to oral health problems can occur, potentially affecting school attendance and performance.

Two recent studies have assessed OHRQoL in children with MIH using the COHIP instrument. In a 2018 study, Hasmun et al. reported that minimally invasive aesthetic treatment of children with incisors affected by MIH can improve their OHRQoL^38^. However, they did not evaluate molars. This reduces the comparability with our study. In a 2022 study, Elhennawy et al. investigated the influence of MIH on OHRQoL in 217 untreated MIH patients and 100 controls, and concluded that MIH had a significant negative impact on children's OHRQoL. Patients with severe MIH had greater negative consequences for their quality of life than patients with mild MIH^39^. These findings are also consistent with our study results. However, the grading of MIH severity was again based on defect size alone, without consideration of tooth sensitivity.

In contrast to the previously mentioned studies, with the exception of Hasmun et al. assessing anterior aesthetics^38^, one of the strengths of the present study is the analysis of patients that had undergone treatment. Both teeth with preventive interventions such as fissure sealants and teeth with restorative treatments in the form of composite restorations were examined. As the included patients had actively presented for treatment at our department and the majority had received some kind of restorative treatment, one may have assumed that this prevents clinical symptoms such as hypersensitivity and masks the impact of more severe forms on OHRQoL. Previous trials have reported pain reduction after the application of composite restorations^40^. Although pre- and postoperative sensitivity could not be compared, hypersensitivity remained a common problem, despite restoration. Moreover, this was not due to widespread restoration failure. Although it has been reported that MIH teeth are often difficult to restore and problems such as reduced resin bond strength occur^41,42^, the estimated survival times of 4.9 and 5.6 years for fissure sealants and composite restorations remained within acceptable boundaries. Composite restoration survival on MIH affected teeth in this study was significantly higher than reported in previous studies^43–45^. This may have been due to the fact that a third of the direct composite restorations were applied over the entire surface of the affected tooth, similar to a direct composite crown, to increase the adhesive surface. Compared to restorations performed due to cariological issues on MIH-free teeth^46–50^, the survival rates of composite restorations on teeth affected by MIH were lower, probably due to above mentioned factors. Therefore, the second null hypothesis has to be rejected.

This study has several limitations. Firstly, its small sample size and exploratory character limit comparisons with other scientific data. The impact of this limitation became evident in our multiple linear regression analysis. Although significant relationships were observed between individual factors and OHRQoL in bivariate analyses, these did not retain their significance in the regression model. This suggests that the limited sample may not have had sufficient power to detect the effects of each predictor within the complex framework of a multivariate analysis. Such an outcome highlights the challenge of discerning the individual contributions of factors like MIH severity, DMFT, dmft, and SCASS to OHRQoL in a setting with constrained sample size. Moreover, as a retrospective and single-center study in a university hospital setting, with patients actively seeking advice and treatment, the sample may not be representative of the general population. This may have biased the results towards a more favorable restorative status and OHRQoL compared to the overall population of children and adolescents with MIH. Moreover, the mostly retrospective analysis of restoration survival did not allow for the standardization of treatment protocols, e.g. the amount of enamel removed, the composite and adhesive material used etc. In light of these limitations, our findings should be interpreted with caution. The divergence between the bivariate analysis and the regression model underscores the need for further research, particularly studies with larger and more diverse samples, to validate and expand upon our results.

## Conclusions

Despite the high rate of restorative treatment with moderate to high long-term survival rates, OHRQoL was reduced in children with MIH, and worsened with the severity of the disease. Many teeth remained sensitive after restoration. The overall survival of composite restorations on teeth affected by MIH was below the survival rates typically reported for composite restorations on MIH-free teeth. The null hypotheses are therefore rejected. Targeted follow-up programs and multicentre strategies for longitudinal ascertainment of these children and adolescent patients should be implemented in order to scientifically monitor this relatively new and prevalent disease.

### Supplementary Information

Supplementary Information.

## Data Availability

Data is available from the corresponding author upon reasonable request.
