# Supplementary material for: Oral health-related quality of life and survival analysis after preventive and restorative treatment of molar-incisor hypomineralisation
Source: Sci Rep. 2024 Jan 8;14:777. doi: 10.1038/s41598-024-51223-3 (PMC10774292; doi:10.1038/s41598-024-51223-3)
Supplement: Supplementary file 1 — Supplementary Information. [file 41598_2024_51223_MOESM1_ESM.docx]

**Appendix**

| **Table A.1** MIH-TNI according to Beker et al. [22,23] | |
| --- | --- |
| Index | Definition |
| Index 0 | No MIH, clinically free of MIH |
| Index 1 | MIH without hypersensitivity, without defect |
| Index 2 | MIH without hypersensitivity, with defect |
| 2a | < 1/3 defect extension |
| 2b | > 1/3 < 2/3 defect extension |
| 2c | > 2/3 defect extension or/and defect close to the pulp or extraction or atypical restoration |
| Index 3 | MIH with hypersensitivity, without defect |
| Index 4 | MIH with hypersensitivity, with defect |
| 4a | < 1/3 defect extension |
| 4b | > 1/3 < 2/3 defect extension |
| 4c | > 2/3 defect extension or/and defect close to the pulp or extraction or atypical restoration |

| **Table A.2** COHIP-19 in English and German according to Sierwald et al. [30] | |
| --- | --- |
| English | German |
| For each statement, please choose the answer that describes you best in the past 3 months regarding your teeth, mouth, or face. | Wie oft in den letzten drei Monaten… |
| 1. Had pain in your teeth/toothache | Hattest du Zahnschmerzen? |
| 1. Had discolored teeth or spots on your teeth | Hattest du verfärbte Zähne oder Flecken auf deinen Zähnen? |
| 1. Had crooked teeth or spaces between your teeth | Hast du festgestellt, dass deine Zähne schief sind oder du Lücken zwischen den Zähnen hast? |
| 1. Had bad breath | Hattest du einen schlechten Atem? |
| 1. Had bleeding gums | Hattest du Zahnfleischbluten? |
| 1. Had difficulty eating foods you would like to eat | Hattest du Schwierigkeiten, wegen deiner Zähne, deines Mundes oder Gesichtes Essen zu essen, das du gerne gegessen hättest? |
| 1. Had trouble sleeping | Hattest du wegen deiner Zähne, deines Mundes oder Gesichtes Schwierigkeiten, zu schlafen? |
| 1. Had difficultly saying certain words | Fiel es dir schwer, bestimmte Wörter auszusprechen aufgrund deiner Zähne, deines Mundes oder Gesichtes? |
| 1. Had difficulty keeping your teeth clean | Hattest du wegen deiner Zähne, deines Mundes oder Gesichtes Schwierigkeiten, deine Zähne sauber zu halten? |
| 1. Been unhappy or sad | Warst du traurig oder betrübt wegen deiner Zähne, deines Mundes oder Gesichtes? |
| 1. Felt worried or anxious | Warst du wegen deiner Zähne, deines Mundes oder Gesichtes aufgewühlt und unbehaglich? |
| 1. Avoided smiling or laughing with other children | Hast du wegen deiner Zähne, deines Mundes oder Gesichtes vermieden, mit anderen Kindern zu lächeln oder zu lachen? |
| 1. Felt that you look different | Hast du das Gefühl gehabt, dass du anders aussiehst aufgrund deiner Zähne, deines Mundes oder Gesichtes? |
| 1. Been worried about what other people think about your teeth, mouth, or face | Warst du besorgt darüber, was andere Leute über deine Zähne, deinen Mund oder dein Gesicht denken? |
| 1. Been teased, bullied, or called names by other children | Wurdest du von anderen Kindern gehänselt, schikaniert oder beschimpft aufgrund deiner Zähne, deines Mundes oder Gesichtes? |
| 1. Missed school for any reason | Hast du die Schule wegen deiner Zähne, deines Mundes oder Gesichtes versäumt? |
| 1. Not wanted to speak/read out loud in class | Wolltest du vor der Klasse nichts sagen oder auch nicht laut vorlesen aufgrund deiner Zähne, deines Mundes oder Gesichtes? |
| 1. Been confident | Warst du selbstsicher wegen deiner Zähne, deines Mundes oder Gesichtes? |
| 1. Felt that you were attractive (good looking) | Hattest du das Gefühl wegen deiner Zähne, deines Mundes oder Gesichtes gut auszusehen? |
| Response categories: “never”, “almost never”, “sometimes”, “fairly often”, and “almost all of the time” | Response categories: “niemals“, “fast niemals“, “manchmal“, “ziemlich oft“, “fast immer“ |

| **Table A.3 Multiple linear regression analysis predicting summary COHIP-19 score** | | | | | | | |
| --- | --- | --- | --- | --- | --- | --- | --- |
| Variable | B | SE | 95% CI for B (Lower) | 95% CI for B (Upper) | β | t-value | p-value |
| Constant | 77.70 | 4.93 | 67.43 | 87.98 |  | 15.78 | <.001 |
| Max. MIH-TNI Code | -1.31 | 0.99 | -3.38 | 0.77 | -0.36 | -1.32 | 0.203 |
| DMFT | 0.24 | 1.45 | -2.78 | 3.26 | 0.04 | 0.17 | 0.870 |
| dmft | 1.58 | 0.79 | -0.06 | 3.22 | 0.35 | 2.01 | 0.058 |
| Max. SCASS Code | -3.28 | 2.01 | -7.48 | 0.92 | -0.35 | -1.63 | 0.119 |
| R² = 0.40, Adjusted R² = 0.28, F(4, 20) = 3.31, p = 0.031 | | | | | | | |

Figure A.1


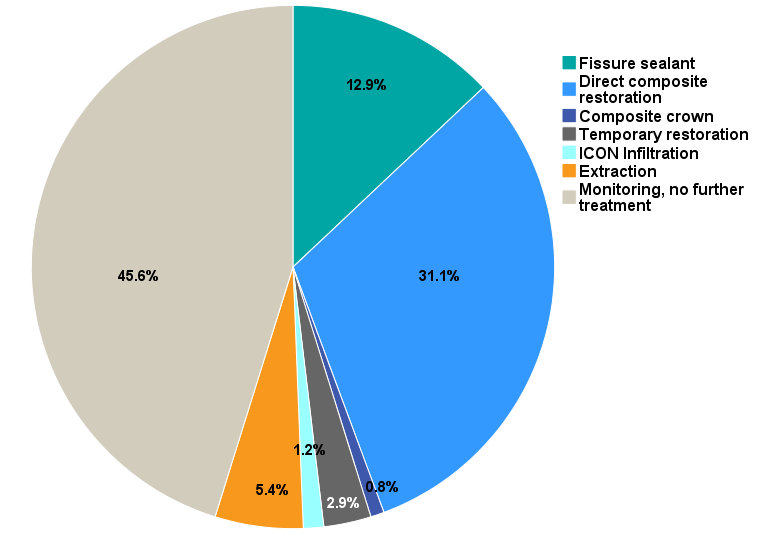


Figure A.1. Treatment of MIH-affected teeth on tooth level
